# The immune landscape of solid pediatric tumors

**DOI:** 10.1186/s13046-022-02397-z

**Published:** 2022-06-11

**Authors:** Shimaa Sherif, Jessica Roelands, William Mifsud, Eiman I. Ahmed, Christophe M. Raynaud, Darawan Rinchai, Abbirami Sathappan, Ata Maaz, Ayman Saleh, Erdener Ozer, Khalid A. Fakhro, Borbala Mifsud, Vésteinn Thorsson, Davide Bedognetti, Wouter R. L. Hendrickx

**Affiliations:** 1 College of Health and Life Sciences, Hamad Bin Khalifa University, Doha, Qatar; 2 Human Immunology Department, Research Branch, Sidra Medicine, Doha, Qatar; 3 Department of Pathology, Leiden University Medical Center, Leiden, The Netherlands; 4 Department of Pathology, Doha, Qatar; 5 Weill Cornell Medicine-Qatar, Doha, Qatar; 6 Deep phenotyping core, Research Branch, Sidra Medicine, Doha, Qatar; 7 Pediatric Hematology and Oncology Division, Sidra Medicine, Doha, Qatar; 8 Department of Pediatrics, Northeast Ohio Medical University, Rootstown, OH USA; 9 Institute of Oncology, Dokuz Eylul University, Izmir, Turkey; 10 Human Genetics Department, Research Branch, Sidra Medicine, Doha, Qatar; 11 Department of Genetic Medicine, Weill Cornell Medicine-Qatar, Doha, Qatar; 12 William Harvey Research Institute, Queen Mary University of London, London, UK; 13 Institute for Systems Biology, 401 Terry Ave N, Seattle, WA 98109 USA; 14 Department of Internal Medicine and Medical Specialties, University of Genoa, Genoa, Italy

**Keywords:** Pediatric cancer, Neuroblastoma, Osteosarcoma, Immune phenotypes

## Abstract

**Background:**

Large immunogenomic analyses have demonstrated the prognostic role of the functional orientation of the tumor microenvironment in adult solid tumors, this variable has been poorly explored in the pediatric counterpart.

**Methods:**

We performed a systematic analysis of public RNAseq data (TARGET) for five pediatric tumor types (408 patients): Wilms tumor (WLM), neuroblastoma (NBL), osteosarcoma (OS), clear cell sarcoma of the kidney (CCSK) and rhabdoid tumor of the kidney (RT). We assessed the performance of the Immunologic Constant of Rejection (ICR), which captures an active Th1/cytotoxic response. We also performed gene set enrichment analysis (ssGSEA) and clustered more than 100 well characterized immune traits to define immune subtypes and compared their outcome.

**Results:**

A higher ICR score was associated with better survival in OS and high risk NBL without MYCN amplification but with poorer survival in WLM. Clustering of immune traits revealed the same five principal modules previously described in adult tumors (TCGA). These modules divided pediatric patients into six immune subtypes (S1-S6) with distinct survival outcomes. The S2 cluster showed the best overall survival, characterized by low enrichment of the wound healing signature, high Th1, and low Th2 infiltration, while the reverse was observed in S4. Upregulation of the WNT/Beta-catenin pathway was associated with unfavorable outcomes and decreased T-cell infiltration in OS.

**Conclusions:**

We demonstrated that extracranial pediatric tumors could be classified according to their immune disposition, unveiling similarities with adults’ tumors. Immunological parameters might be explored to refine diagnostic and prognostic biomarkers and to identify potential immune-responsive tumors.

**Supplementary Information:**

The online version contains supplementary material available at 10.1186/s13046-022-02397-z.

## Background

Cancer is one of the leading causes of death in children worldwide, and the recorded incidence tends to rise with time [1]. In the US [2], and in other high-income countries [3], cancer is the leading cause of death by disease past infancy among children.

The overall incidence rates of childhood cancer vary between 50 and 200 per million children across the world [1]. The most common categories of childhood cancers include leukemias, brain tumors, lymphomas, neuroblastoma and nephroblastoma (Wilms tumor, WLM) [4]. Solid tumors comprise almost half of the cancer cases [5]. Neuroblastoma (the most frequent pediatric extra-cranial tumor) and WLM are tumor types that occur almost exclusively in children [6].

Although major progress has been made in the treatment of pediatric cancers since the 1960s, and despite the intensification of treatment, contemporary progress has been limited or absent [7–9]. For those where the disease relapses, survival remains poor and for patients presenting with non-invasive tumors, surgical intervention can cause major long-term consequences. In addition, many children with high-risk cancers experience severe, life-threatening, or fatal drug toxicity during their medical treatment [10].

The implementation of immune checkpoint inhibitors that unleash natural anti-tumor immunity has changed the therapeutic scenario of cancer patients, especially in adults [11]. One of the major factors associated with responsiveness to immune checkpoint inhibition is the availability of neoantigens, which is the function of the total number of non-synonymous mutations (tumor mutational burden) [9, 12]. Pediatric cancers display overall a low mutational burden [13] and not surprisingly, the activity of immune checkpoint inhibitors in this setting has been extremely limited [14]. In this respect, the FDA, for instance, has approved the use of checkpoint inhibition (PD1 blockades) for the treatment of children with mismatch repair deficient, microsatellite unstable, or hypermutated tumors [15], independently of the tumor histology. However, immune-mediated cancer cell recognition and killing might occur independently of the availability of mutated antigen, and other forms of immunotherapy might be exploited in tumors with low mutational load [16], including pediatric cancers. These include for instance T-cell or NK-cell based adoptive therapy [17], vaccines directed at non-mutated antigens, and oncolytic therapy, alone or in combination with checkpoint inhibitors [18] [19]. These approaches have demonstrated encouraging results in pre-clinical models [17–19], but clinical successes using immunotherapy have been only obtained in neuroblastoma, a tumor type for which observed spontaneous remissions are likely mediated by cellular immunity [20, 21]. In high-risk patients, complementing standard therapy with dinutuximab, which targets the NBL-associated antigen GD2, interleukin-2 (IL-2) and granulocyte-monocyte colony-stimulating factor (GM-CSF) increased event-free and overall survival [22] but is not curative for the majority of patients who will ultimately relapse and die [17].

In parallel with the results of the activity of immune checkpoint inhibition, studies in adult patients affected by solid tumors have shown that the density, location, and functional orientation of the immune infiltrate influence the risk of relapse and death after tumor resection [16, 23]. Notably, in colon cancer for instance, a robust and protective intratumoral cytoxic T-cell response can occur in absence of a high mutational load [24, 25]. In addition, within tumor types, the associations between intratumoral T-cell infiltration and mutational load is in general weak or absent [26, 27], and mostly driven by extreme cases such as the heavily hypermutated tumors (mismatch-repair-deficient/microsatellite unstable tumors) [28]. In fact, both T-cell cytotoxic signature and tumor mutational load are independently associated with better response to immunotherapy [29].

One of the signatures capturing a favorable T-cell/cytotoxic anti-tumor immune response is the Immunologic Constant of Rejection (ICR) [30–32]. The ICR consists of 20 genes that reflect activation of Th1 signaling (IFNG, TXB21, CD8B, CD8A, IL12B, STAT1, and IRF1), expression of CXCR3/CCR5 chemokine ligands (CXCL9, CXCL10, and CCL5), cytotoxic effector molecules (GNLY, PRF1, GZMA, GZMB, and GZMH) and compensatory immune regulators (CD274/PD-L1, PDCD1, CTLA4, FOXP3 and IDO1) [28, 33]. The ICR has been associated with better prognosis in the breast cancer [33, 34], in which it could further stratify high-risk patients defined by standard prognostic signatures used in the clinical practice [35]. Added prognostic value has also been observed in a large meta-analysis of gene expression data from patients with sarcoma [36]. Moreover, ICR modules have been associated with response to checkpoint inhibitions and other forms of immunotherapy, including adoptive therapy [37], vaccine therapy [38], and local immunotherapy (Imiquimod) [39]. Overall, ICR modules have been described in the large majority of classifiers developed in the context of immune checkpoint studies [40]. Large pan-cancer analyses of adult solid tumors by the TCGA have defined conserved modules driving discrete immune subtypes associated with differential survival, adding granularity to the described association between T-cell infiltration and favorable prognosis [41]. Moreover, metrics capturing the balance between activation of specific oncogenic pathways and immunologic signals could further used to increase the prognostic and predictive values of individual signatures [28, 42, 43].

A better understanding of the biology of childhood cancers, including the relationship between cancer cells and the immune system, is of paramount importance for the development of more effective therapeutic approaches and stratification systems in this setting.

Here, we performed a pan-cancer analysis pediatric solid tumors using transcriptomic data in the TARGET dataset. Specifically we tested the prognostic impact of the ICR and explored the existence of different immune subtypes, as previously proposed in the context of adult solid tumors [41].

## Methods

All analysis was done in R version 3.6.1, software names are R packages unless stated otherwise.

### Data acquisition and normalization

RNA-seq data for 5 pediatric tumors: Wilms tumor (WLM), neuroblastoma (NBL), osteosarcoma (OS), rhabdoid tumor (RT) and clear cell sarcoma of the kidney (CCSK) from the TARGET pediatric dataset, which is published on the GDC portal website, were downloaded and processed using TCGAbiolinks (v. 2.14.1) [44]. Gene symbols were converted to official HGNC symbols using TCGAbiolinks, genes without symbol or gene information were excluded and this resulted in a pan-cancer expression matrix with 20,155 genes. Metastatic tumor, recurrent primary tumor or blood derived samples were excluded, and a single primary tumor (TP) sample was analyzed for each patient.

RNA-seq gene counts were normalized using the TCGAanalyze_Normalization function from TCGAbiolinks, including within-lane normalization procedures to adjust for GC-content effect, between-lane normalization procedures to adjust for distributional differences between lanes as sequencing depth and quantile normalized using TCGAbiolinks. After normalization, the pan-cancer matrix was split per cancer type and log2-transformed. The clinical data of the TARGET study was obtained from the GDC portal.

### ICR classification

The gene expression data of the ICR signature used to cluster the patients from each cancer type and pan-cancer using the ConsensusClusterPlus (v.1.42.0) [45] with the following parameters: 5000 repeats, a maximum of six clusters and agglomerative hierarchical clustering with Ward.D2 criterion. The optimal number of clusters was determined based on the Calinski-Harabasz index.

The three obtained clusters were annotated as ‘ICR High’, ‘ICR Medium’ and ‘ICR Low’, where ‘ICR High’ showed the highest expression of ICR genes and ‘ICR Low’ the lowest. ICR score was calculated for each sample, defined as the mean of the normalized, log2-transformed gene expression values of the ICR genes. Heatmaps were drawn using the ComplexHeatmap (v.2.6.2) [46].

T-distributed stochastic neighbor embedding (t-SNE) plot was used as a dimension reduction technique on the complete RNA expression matrix using Rtsne (v. 0.15) [47] (settings perplexity = 15, theta = 0.5). t-SNE plots were annotated for cancer types and ICR clusters.

### Survival analysis

Clinical files contain survival data and clinical parameters such as age at diagnosis, tissue type, vital status, disease stage and disease metastasis, amongst others. For the overall survival analysis, we used the time to death and time to last follow up, vital status. For the event free survival, we considered; 1) relapse, 2) progression, 3) second malignant neoplasm death, and 4) death without remission, as events. We performed survival analysis and plotted the Kaplan-Meier curves using the ggsurv function from survminer (v. 0.4.8) [48]. Patients with less than 1 day of follow-up were excluded and survival data were censored after a follow-up duration of 10 years. Hazards Ratio (HR) between ICR Low and ICR High groups or between the six immune subtypes and the corresponding *p*-values were calculated using X^2^ test. Confidence intervals (97.5% CIs) were defined using survival (v. 2.41–3) [49].

Cox proportional hazards regression analysis was performed using Survival and visualized as a forest plot. Cancer types were added as a factor in the multivariate analysis. We applied the cox.zph function, to test the proportional hazards assumption (PHA). The same method was used to correct for the clinical parameters that contribute to the survival across immune subtypes in the high risk NBL without MYCN amplification cohort. These clinical parameters are the (Mitosis-Karyorrhexis Index) MKI (High, Intermediate and Low), Ploidy (diploid and hyperploid), Age group (0-18 m,18 m-5y and above 5y). Forest plots were generated using forestplot (v.1.7.2) [50].

### Immune cell subpopulation and oncogenic pathway enrichment analysis

To determine the enrichment of particular gene sets, that reflect either immune cell types or certain oncogenic pathways, we performed single sample gene set enrichment analysis (ssGSEA) on the log2-transformed, normalized gene expression data using GSVA (v.1.38.2). Immune cell-specific signatures were used as described in Bindea et al. [51] with slight modification. The dendritic cell (DC) signature was replaced by immature dendritic cells (iDC), plasmacytoid dendritic cells (pDC), myeloid dendritic cells (mDC) and DC. Additionally, the regulatory T-cell (Treg) signature was used as described in Angelova et al. [52]. Gene sets that reflect specific tumor-related pathways were selected from multiple sources as described in detail in [33, 53, 54], and gene sets reflecting cancer related immune signatures were used as previously described by Thorsson et al. [41] .The association between continuous gene set enrichment scores and survival was calculated as described above. Differences between the HRs of signatures were illustrated in a heatmap (ComplexHeatmap (v. 2.2.0)) and the *p*-values were calculated by the cox formula. Signatures with a *p* value > 0.1 across all tumors were excluded.

### Comprehensive pediatric immune subtypes

ssGSEA was performed using 105 of the 108 previously described immune signatures [41]. Three signatures were excluded from the analysis due to missing gene expression information. Spearman correlation between the resulting enrichment scores was calculated and visualized using corrplot (v.0.90). Signature modules were identified visually and then patients were clustered according to the ssGSEA enrichment of the 5 signatures representing the identified modules, previously identified by Thorsson et al. Sample clustering was carried out using k-means clustering (km = 6, repeats = 10,000), using ComplexHeatmap. The gap statistics was used to calculate the optimal number of clusters.

Stacked bar chart from ggplot2 (v. 3.3.3) was used to show the percentage of each cancer type in the immune subtypes and the percentage of each immune subtype within each cancer type. Density plots from ggplot2 were used to show the median of enrichment scores of selected immune signatures from the 105 signatures [41], in addition to the log2 values of *HLA-1* and *HLA-2* from the filtered normalized RNAseq matrix.

### Gene expression correlation

Correlation matrices of ICR genes expression were generated by calculating the Pearson correlation of the ICR genes’ expression within cancer types and pan-cancer using the corrplot (v.0.90), CCSK was excluded from this correlation analysis because of the small sample size (*n* = 13). Spearman correlation was performed on the enrichment matrix of 105 tumor immune expression signatures [41] and plotted using corrplot (v. 0.84). Correlation matrices of NK-cells / CD8T enrichment scores and the enrichment score (ES) of selected oncogenic pathways were calculated using Pearson correlation and plotted by ComplexHeatmap.

### Immune checkpoints expression

We used a list of immune checkpoints, divided into activating and inhibitory. The median values of the log2 transformation of the normalized gene expression counts of these genes were used and plotted by ComplexHeatmap.

### CIBERSORTx immune cells fractions

In order to compare the immune cell fractions between different immune subtypes, we analyzed the normalized gene expression data of the 408 pediatric samples using the CIBERSORTx website. The relative proportions of 22 immune cell types within the leukocyte compartment (LM22) were estimated. Cell fractions were visualized in barcharts and boxplots using ggplot2. We summed the proportions of related immune cells together in ‘Aggregates’ to facilitate comparisons [41]. Lymphocytes are the sum of B-cells naive, B-cells memory, T-cells CD4 naive, T-cells CD4 memory resting, T-cells CD4 memory activated and T-cells follicular helper, T-cells regulatory, Tregs, T-cells gamma delta, T-cells CD8, NK-cells resting, NK-cells activate and Plasma cells fractions. Macrophages are the sum of Monocytes, Macrophages M0, Macrophages M1 and Macrophages M2 fractions. Dendritic cells are the sum of Dendritic cells resting and Dendritic cells activated fractions. Mast cells are the sum of Mast cells resting and Mast cells activated fractions.

## Results

### The prognostic value of ICR differs across pediatric cancer types

**Fig. 1:**
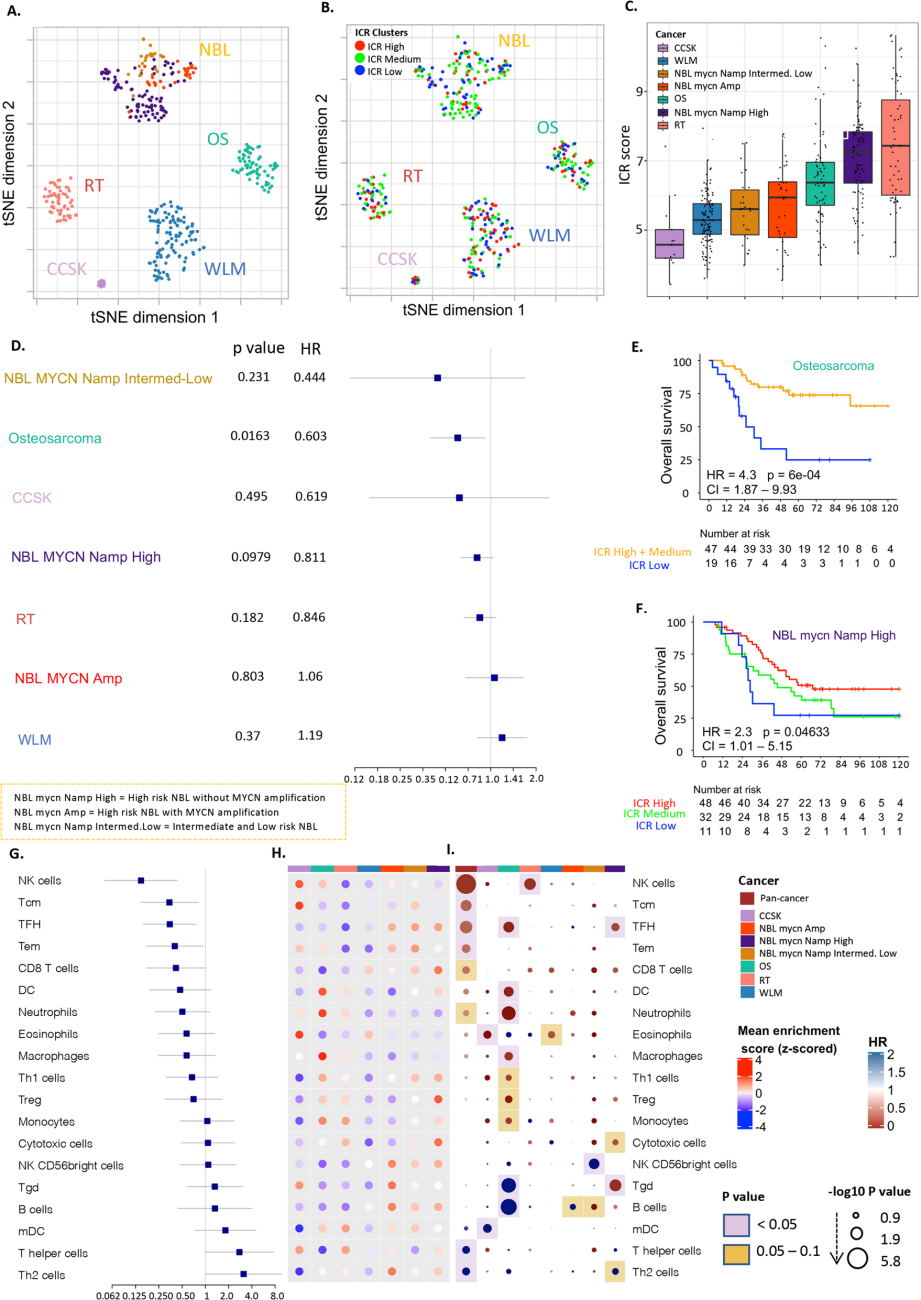
Prognostic role of immune signatures in pediatric tumors. **A** tSNE plot of filtered normalized expression values annotated by pediatric cancer types. **B** tSNE plot of filtered normalized expression values annotated by ICR clusters. **C** Boxplot of the ICR score across pediatric tumor types. **D** Forest plot of the association of continuous ICR scores with survival across tumors (**E)** Kaplan-Meier overall survival curve for ICR High + medium (orange) versus ICR low (blue) in Osteosarcoma. **F** Kaplan-Meier overall survival curve for ICR High versus ICR low in high risk NBL without MYCN amplification. **G** Forest plot of Hazards ratio of immune subpopulations across different pediatric tumors. **H** Heatmap of enrichment scores of immune cells signatures. **I** Hazards ratio heatmap of these signatures, the color of the circle representing the HR; HR below 1 is red and above 1 is blue, the radius size representing the -log10 *p* value; Larger size has higher -log10 p value and more significant association with survival, the color of the background corresponding to the p value; if pink; p value is less than 0.05, if yellow; p value between 0.05 and 0.1 and the white means p value above 0.1

We analyzed the expression profiles of patient samples from five distinct solid pediatric cancer types: WLM, NBL, OS, RT and CCSK from TARGET dataset (https://ocg.cancer.gov/programs/target). After exclusion of the following patients: 20 OS patients, who were older than 18 years-old; one NBL patient, who did not have MYCN status information; one RT patient, whose sample clustered with NBL samples based on the whole transcriptome; we analyzed 408 patient samples (WLM (*n* = 118), NBL (*n* = 150), OS (*n* = 68), RT (*n* = 59), CCSK (*n* = 13)). The NBL cohort was separated into three groups based on the annotated COG (Children’s Oncology Group) risk group and the MYCN gene amplification status: high risk NBL with MYCN amplification (*n* = 33), high risk NBL without MYCN amplification (*n* = 91), and Intermediate and low risk NBL (NBL-ILR) (*n* = 26), because these subgroups were shown to have distinct immune infiltration [55, 56]. Dimension-reduction using t-Distributed Stochastic Neighbor Embedding (tSNE) based on the whole transcriptome also showed separation of NBL subgroups (Fig. 1A),(Wei et al. 2018) therefore, we considered each subgroup as a separate cancer type in our analysis.

We used the expression of ICR genes to characterize the degree of the Th1/cytotoxic intratumoral response. The ICR genes exhibited high overall correlation with each other in most TARGET pediatric solid tumor cohorts, with a lower correlation in high risk NBL with MYCN amplification and in WLM (Supplementary Fig. 1). Samples from each cancer type were separated into three ICR clusters (“ICR High”, “ICR Medium” and “ICR Low”) (Supplementary Fig. 2A). While dimension reduction of the expression data shows no clear segregation of samples by ICR clusters within each tumor type (Fig. 1B), a clear difference in the distribution of ICR scores across cancer types was observed (Fig. 1C). Lower ICR scores were found in WLM and CCSK, while RT had the highest ICR scores. Significant differences in ICR scores were observed across NBL subgroups (*p* < 0.00001) for high risk NBL without MYCN amplification vs Intermediate and low risk NBL and high risk NBL with MYCN amplification respectively (Supplementary Fig. 2B), reflecting large immune orientation differences between samples within NBL. Substantially lower ICR scores were observed in high risk NBL with MYCN amplification and in intermediate and low risk NBL when compared to high risk NBL without MYCN amplification. This finding is consistent with previous reports of poor T-cell infiltration in high risk NBL with MYCN amplification [56, 57] [55] and higher T-cell infiltration in high risk NBL without MYCN amplification [56]. Overall survival analysis of continuous ICR scores showed significant association of ICR scores with high survival rate in Osteosarcoma (*p* < 0.016) (Fig. 1D) and comparing the survival between the ICR clusters showed that in Osteosarcoma (OS), the ICR Low group had significantly lower overall survival (*p* < 0.001) (Fig. 1E) and event-free survival estimates (*p* < 0.05) (Supplementary Fig. 2D) compared to the other groups combined. The same pattern was found in high risk NBL without MYCN amplification where ICR High was associated with better overall survival compared to ICR Low (Fig. 1F). This pattern was reversed in WLM (Supplementary Fig. 2C), as has been observed in adult kidney tumors [28]. No significant association with survival was found in rhabdoid tumor or clear cell sarcoma of kidney (Kaplan Meier curve for CCSK not showed because of the low number of samples *n* = 13).

Since ICR was most prognostic in Osteosarcoma and high risk NBL without MYCN amplification, we proceeded to examine which tumor intrinsic attributes correlate with immune infiltration, reflected by ICR score, in OS and high risk NBL without MYCN amplification. Tumor intrinsic pathways that correlated with ICR score in these tumors (Supplementary Fig. 3A) are TNFR1, PI3K Akt mTOR signaling, Immunogenic cell death, Apoptosis, mTOR and others, while signatures inversely correlated with survival in both tumors include barrier genes, mismatch repair, proliferation, G2M checkpoints. Wnt/beta-catenin signaling showed very strong inverse correlation with ICR in Osteosarcoma but not in high risk NBL without MYCN amplification (Supplementary Fig. 3A).

We then examined the association of tumor intrinsic attributes with survival in these tumors, Wnt/beta-catenin pathway was significantly associated with a worse prognosis in OS (*p* < 0.05) (Supplementary Fig. 3C). We did not observe this same association in high risk NBL without MYCN amplification (Supplementary Fig. 3C). In this neuroblastoma subgroup, several pathways were associated with worse prognosis, such as Myc targets, Glycolysis, mTORC1, DNA repair, Mismatch repair, E2F targets, G2M checkpoints and proliferation (Supplementary Fig. 3C).

### The functional orientation of infiltrating immune cells influences the clinical outcome of pediatric cancers

To explore the different immune characteristics of pediatric tumor types in more depth, we compared the enrichment of leukocyte subpopulations within and among cancer types (pan-cancer), using the gene expression signatures of previously published datasets [51, 52] (Supplementary Table 1), as described in the methods section. Signatures such as NK-cells, Tcm, TFH, Tem, CD8+ T-cells and neutrophils were significantly associated with better overall survival in the pan-cancer analysis, while T helper and Th2 cells were associated with worse prognosis (Fig. 1G, Supplementary Table 2).

Compared with other cancer types, Osteosarcoma showed an immune active phenotype illustrated by increased mean enrichment of transcripts for dendritic cells (DC), macrophages, neutrophils, and mDC (Fig. 1H). Enrichment scores for some leukocyte populations were associated with significantly improved prognosis as TFH, DC, neutrophils, macrophages, monocytes, Th1 and regulatory T cells (Treg) (Fig. 1I), while B cell and gamma delta T-cell enrichments were associated with significantly worse survival in this cancer type (Fig. 1I).

In Neuroblastoma, T-cells, CD8+ T cells, Th17, NKT cells, Th1 cells, Treg cells, and DCs were significantly higher enriched in the high risk NBL without MYCN amplification group compared to high risk NBL with MYCN amplification (*p* < 0.05), TFH was high in the 3 subgroups and showed significant positive association with survival in the high risk NBL without MYCN amplification group. Gamma delta T cell (Tgd) enrichment also showed high association with survival in high risk NBL without MYCN amplification group (*p* < 0.05). However, Th2 cells and NK CD56 bright cells were significantly higher enriched in the high risk NBL with MYCN amplification group compared to high risk NBL without MYCN amplification group (*p* < 0.05) and in intermediate and low risk NBL, a strong association of NK CD56 bright cells with worse prognosis was seen (*p* < 0.05).

In kidney tumors, WLM and CCSK were characterized by low immune infiltration illustrated by a low ICR score, while RT showed the highest ICR score (Fig. 1B). Decreased infiltration was associated with better survival in WLM (Supplementary Fig. 1C); this reverse association was previously observed in the adult kidney cancer [28]. Overall low enrichment of immune subpopulations was observed in WLM with no clear association with survival (Fig. 1H, I).

In pan-cancer, expression patterns consistent with enrichment of several immune cells were associated with favorable prognosis, including NK-cells, Tcm, TFH, Tem, CD8 T cells and Neutrophils, while the pattern was reversed in Th2 and T helper cells, consistent with similar observations in adult cancer. However, due to the small sample size of some cohorts, we could not identify consistent significant prognostic biomarkers in the leukocyte populations across all cancers.

### Identification of distinct immune subtypes of pediatric tumors

**Fig. 2:**
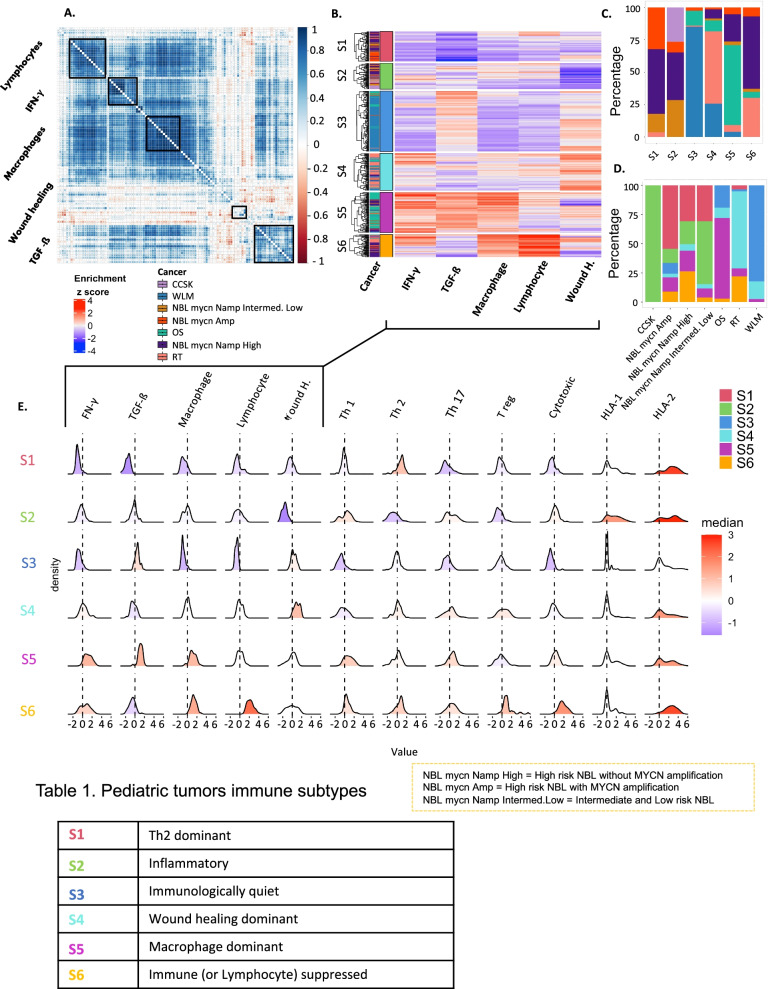
Immune subtypes of pediatric tumors. **A** Spearman’s correlation of 105 cancer immune signatures. Highly correlated signatures are clustered into 5 modules (black rectangles). **B** Heatmap showing the enrichment of immune signatures for each patient. Tumors are clustered into 6 subtypes based on the enrichment patterns. **C** Distribution of the cancer types within the immune subtypes. Colors represent the cancer types. **D** Distribution of the immune subtypes within TARGET pediatric tumors. Colors represent the immune subtypes. **E** Distributions of signature scores within the six immune subtypes (rows), with dashed line indicating the median

To further elucidate the impact of the cancer immune phenotypes in pediatric solid cancer, we expanded our analysis to a collection of previously published immune signatures. We performed ssGSEA on 105 immune signatures and clustered them to define modules of highly correlated immune signatures (Supplementary Table 3). We identified 5 main clusters of signatures (5 modules). Interestingly, in each of these modules we could identify one of the representative signatures presented in Thorsson et al. [41] (IFN-γ, TGF-ß, Macrophages, Lymphocytes, Wound healing) (Fig. 2A). This finding demonstrates that modules of immune signatures in pediatric cancer showed a similar pattern of correlation as those identified in adult solid tumors, reflecting the robustness of these modules.

We then clustered the 408 patients based on the enrichment scores of these 5 representative immune gene signatures, into 6 immune subtypes (S1 to S6) with distinct immunologic orientations (Fig. 2B). Each subtype includes patients from several tumor types (Fig. 2C), and each tumor type consists of different immune subtypes (Fig. 2D). We generated density plots of each of the five representative immune signatures and of each of seven additional immune biomarkers that are known to reflect the immune orientation (Fig. 2E). This allowed us to better interpret each immune subtype and label them based on their enrichment profiles (Table 1). S1 is referred to as Th2 dominant subtype as it has the highest Th2 and the lowest TGF-ß, Macrophage, Lymphocyte and IFN-γ signal. S2 was labeled the Inflammatory subtype since it exhibits the highest Th1-Th2 ratio, the highest HLA1 expression and lowest wound healing enrichment. Since high TGF-ß stands out in S3, in addition to the low enrichment of Th1, Th17 we call this subtype Immunologically quiet. S4 or the Wound healing subtype is dominated by the highest wound healing enrichment, shows high Th2 and Treg cells presence. The S5 subtype has increased TGF-ß and IFN-γ, ICR signatures, high Th1 and Th17 enrichment but seems to be immunologically impaired by high Macrophage presence and is thus referred to as Macrophage dominant. The last immune subtype, S6 or the Lymphocyte suppressed subtype, has an enrichment of almost all properties of a high immune infiltration (highest ICR) including counter-regulatory signals from Th2, Treg, downregulated HLA1, and Macrophage presence. However, it is also characterized by high expression of immune checkpoints and exhaustion markers, so we call it immune (or lymphocyte) suppressed (Fig. 2E).

We compared the immune cell fractions across the immune subtypes using CIBERSORTx (Supplementary Fig. 5, Supplementary Fig. 6). High proportions of macrophages were observed in S5, with increased M2 macrophages proportions in S4, high proportions of mast cells observed in S3 and elevated proportions of lymphocytes were found in S2 which has the highest survival. In addition, it was very clear the high proportions of lymphocytes in S6 that characterized by the elevation of immune checkpoints and exhaustion markers which suppress the effect of T lymphocytes and make it in an exhaustion status (Fig. 4B).

To further understand how each immune subtype contributes to the overall immune response for each tumor type we generated the heatmaps in the Supplementary Fig. 7. The Rhabdoid tumor for example is mainly dominated by the S6, S5 and S4 immune subtypes, which are characterized by the highest ICR enrichment scores. This in turn contributes to the high ICR scores observed in the RT. The Immune suppressed S6 subtype is characterized by the enrichment of almost all properties of a high immune infiltration in addition to the signals from Th2, Treg, downregulated HLA1, Macrophage presence, immune checkpoints and exhaustion markers as previously demonstrated. While S5 (Macrophage dominant) is suppressed by very high M2 macrophage presence. Finally, S4 is suppressed by the increased expression of wound healing genes. This explains why a high ICR score is not associated with a better prognosis in these RT patients.

### Immune subtype classification segregates tumors into distinct risk categories

**Fig. 3:**
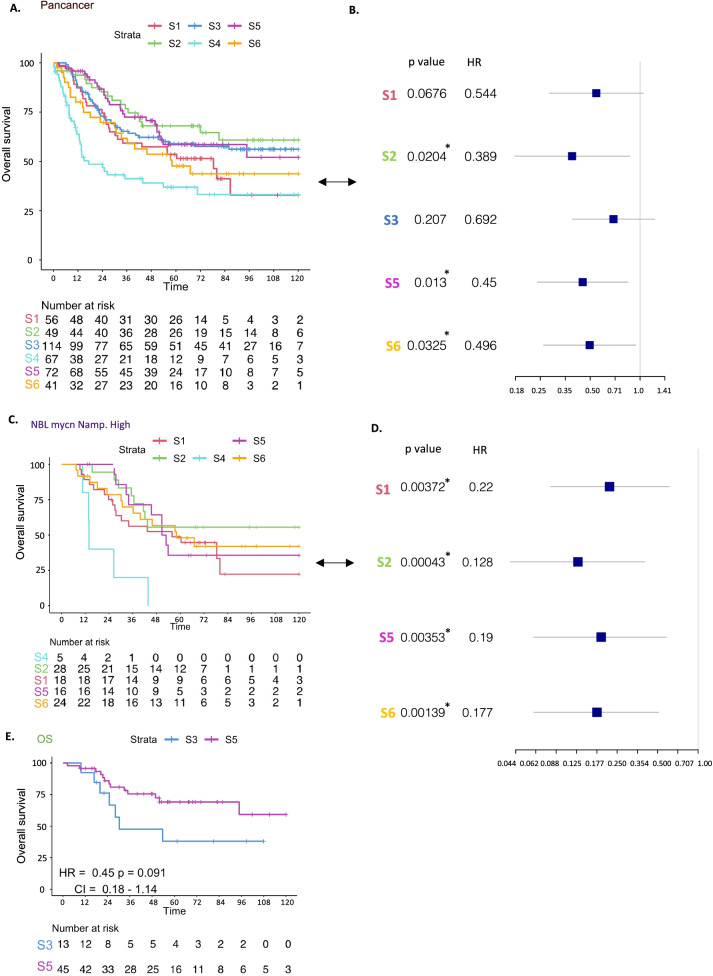
Overall survival across Immune subtypes. **A** Kaplan-Meier overall survival curve for immune subtypes. **B** Forest plot showing HRs (overall survival) of immune subtypes; S1, S2, S3, S5, S6 versus S4, and p value corrected for cancer types. **C** Kaplan-Meier overall survival curves for immune subtypes within tumor types for high risk NBL without MYCN amplification. **D** Forest plot showing HRs (overall survival) of immune subtypes within high risk NBL without MYCN amplification; S1, S2, S5, S6 versus S4. **E** Kaplan-Meier overall survival curves for immune subtypes within Osteosarcoma

Cox proportional hazard models demonstrated significant violation of the model when adding the cancer type as a covariate, so we stratified the model for the cancer type and performed Cox proportional hazard regression analysis which showed significant differences in overall survival between subtypes (Fig. 3A). The best prognosis was observed for the Inflammatory subtype (S2) while the subtype with the worst survival was the Wound healing dominant subtype (S4). The lowest enrichment of Wound healing signature was observed in S2, the subtype with the best survival among other immune subtypes (Fig. 3A), suggesting an association of Wound healing signature expression with prognosis in pediatric tumors. In S2, in addition to the low enrichment of Wound healing, high Th1 and low Th2 infiltrations were observed, while the reverse (high Th2 and low Th1 infiltration) (Fig. 2E) was observed in S4. This observation corroborates the favorable prognostic role of a Th1 orientation of the tumor microenvironment in this setting. In order to assess whether the difference in survival across immune subtypes is due to the tumor type distribution between clusters, we performed a multivariate analysis using a Cox proportional hazards model including the cluster (immune subtype) and cancer type as co-variants, and we again found a significant difference in survival between S2 and S4 (*p* = 0.02), between S5 and S4 (*p* = 0.013), and between S6 and S4 (*p* = 0.0325), demonstrating the prognostic impact of this immune stratification (Fig. 3B).

In order to test the prognostic value of our immune stratification within each tumor type, we compared overall survival between immune subtypes within each tumor (Fig. 3C-E, Supplementary Fig. 4). Interestingly, for high risk NBL without MYCN amplification tumors, we found significant differences between all the immune subtypes versus S4 (*p* < 0.05) which indicates the presence of subgroups with distinct immunological features within the high risk NBL without MYCN amplification cohort. The same survival pattern was observed for S4 in both Wilms and Rhabdoid tumors, and a clear difference in survival between the S3 and S5 subtypes were seen in Osteosarcoma (*p* = 0.09) (Fig. 3E). For CCSK the number of samples was too small and therefore the K-M curve was not plotted. These observations highlight the immune heterogeneity within tumors and the importance of understanding the immunological features of pediatric tumors and their subgroups in order to raise the therapeutic effect.

NBL is a heterogeneous tumor and different clinical parameters contribute to the survival of NBL [56, 58], as previously mentioned, a significant difference across immune subtypes within the high risk NBL without MYCN amplification was observed (Fig. 3C, D, Supplementary Table 4), we performed multivariate analysis to correct for the contribution of other clinical parameters in the survival of NBL, a significant difference in survival was found between S2 (*p* = 0.0319) and S6 (*p* = 0.0452) compared to S4. Similarly, we evaluated subsetting other cancer types based on different clinical parameters, however, no meaningful results were found. (Figshare: 10.6084/m9.figshare.19731910).

### Immune checkpoints expression pattern varies across different immune subtypes

**Fig. 4:**
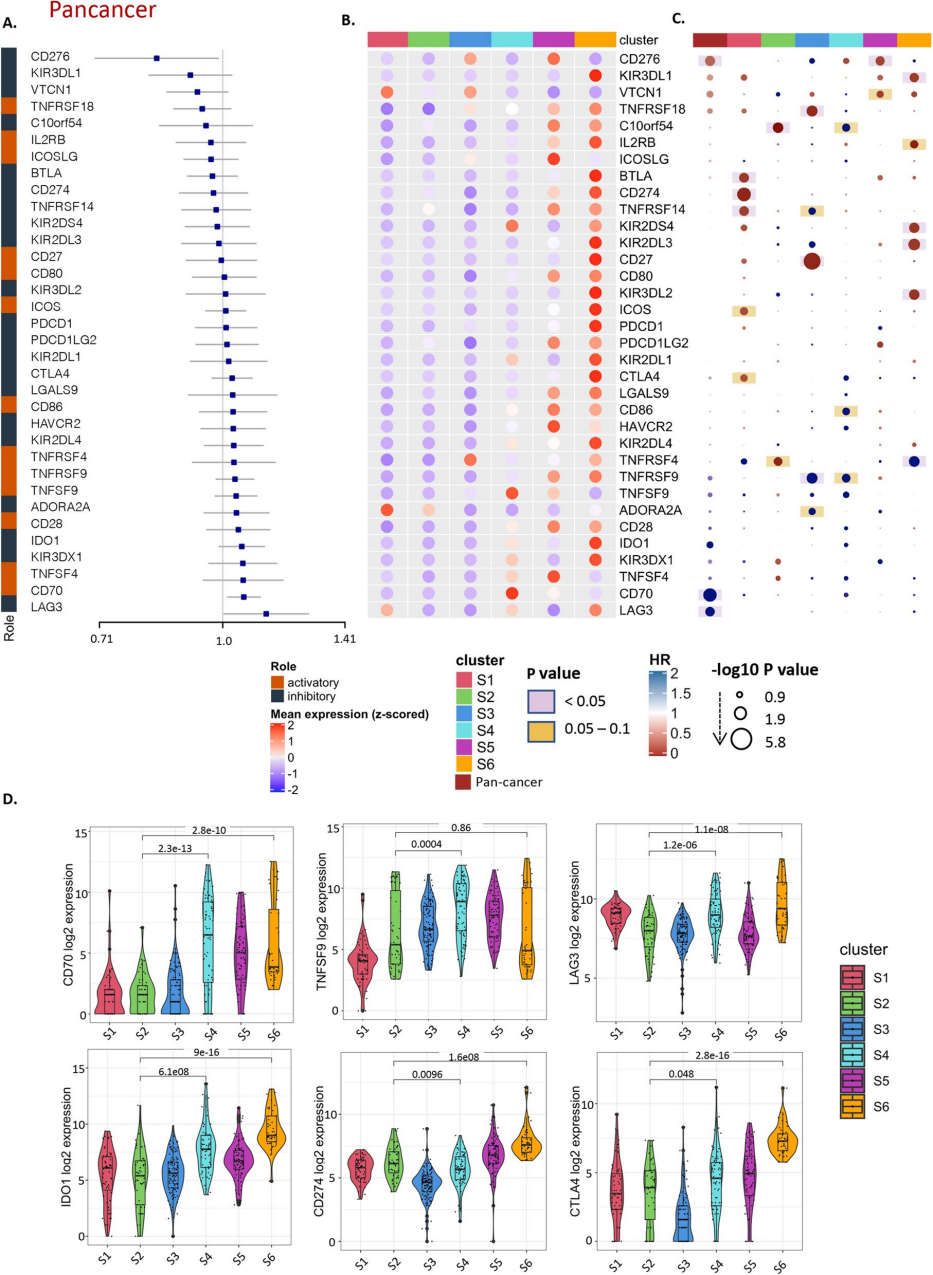
Immune checkpoints expression across Immune subtypes. **A** Pan-cancer Forest plot showing HRs (overall survival) and *p*-values of immune checkpoint expression. **B** Heatmap of log2-transformed expression of immune checkpoints aggregated by the median for each immune subtype, split into activator and inhibitor and annotated by immune subtypes. **C** Hazard ratio heatmap of these immune checkpoints, the color of the circle representing the HR; HR below 1 is red and above 1 is blue, the radius size representing the -log10 p value; Larger size has higher -log10 p value and more significant association with survival, the color of the background corresponding to the p value; if pink; p value is less than 0.05, if yellow; p value between 0.05 and 0.1 and the white means p value above 0.1. **D** Violin plots showing the log2 expression of immune checkpoints across the different immune subtypes

To understand the prognostic role of immune checkpoints in pediatric tumors, we performed survival analysis for checkpoint expression pan-cancer and across our immune subtypes. The *CD276* gene was significantly associated with survival in pan-cancer analysis (Fig. 4A, Supplementary Table 5). We noticed that immune checkpoints that are strongly associated with better prognosis as *CD276*, *KIRD3DL1, VTCN1, C10orf54 (VISTA),* are low enriched in S4 (Fig. 4B), while those associated with worse prognosis as *LAG3*, *CD70*, TNFSF4, IDO1, *KIRD3DX1, CD28 and TNFSF9*, were highly expressed in S4. To deeper understand the prognostic effect of the immune checkpoints’ expression within each immune subtype we generated an HR heatmap annotated by immune subtypes (Fig. 4C). In S4 a unique pattern of association with a worse prognosis was found with *C10orf54 (VISTA)* and *CD86* (p 0.05–0.1). While *TNFRSF9* was associated with worse survival in S4 and S3. In pan-cancer significant association of *CD70* and *LAG3* expression with poor survival was observed (*p* < 0.05). Some immune checkpoints show a reverse pattern of survival with different subtypes as *C10orf54* that associated with reverse favorable prognosis in S2, *TNFRSF14* across S1 and S3 and *TNFRSF4* across S2 and S6. These findings reflect the variation in the prognosis of immune checkpoints expression within different tumor types.

High expression of immune checkpoints was observed in the Leukocyte dominant subtype (S6) compared to other immune subtypes (Fig. 4B, D). This could be explained by the exhaustion status seen in this immune subtype which displays the highest enrichment of lymphocytes (Fig. 2E).

### Activation of oncogenic pathways is associated with the differential immune disposition

**Fig. 5:**
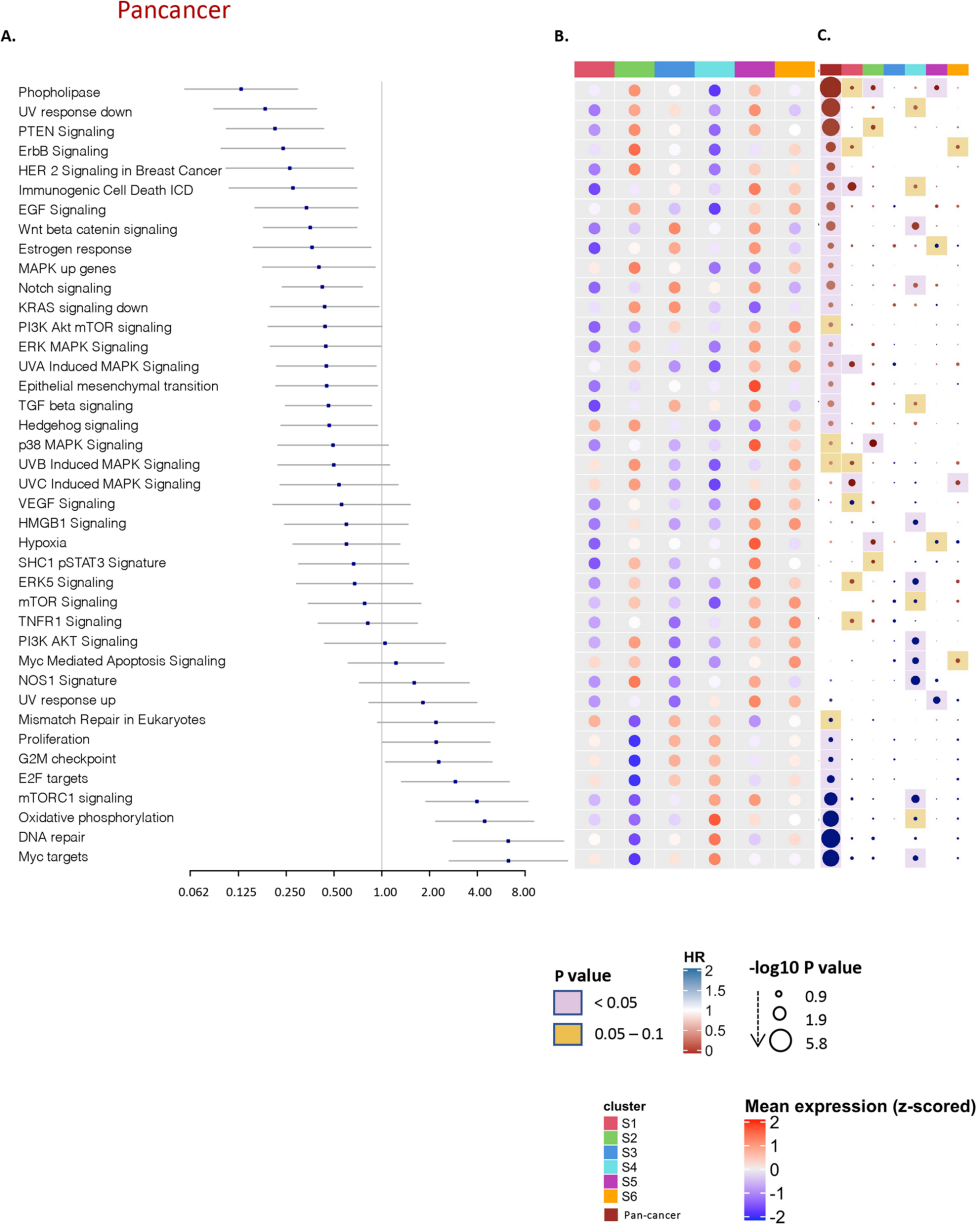
Intrinsic selected oncogenic pathways across immune subtypes. **A** Pan-cancer forest plot showing HRs (overall survival) and p-values of selected oncogenic pathway enrichment scores. **B** Heatmap of the enrichment scores of selected oncogenic pathways, blue colors corresponding to lower enrichment and the red for the high enrichment scores, annotated by the 6 immune subtypes and pan-cancer. **C** Hazards ratio heatmap of these pathway enrichment scores, the color of the circle representing the HR; HR below 1 is red and above 1 is blue, the radius size representing the -log10 *p* value; Larger size has higher -log10 *p* value and more significant association with survival, the color of the background corresponding to the *p* value; if pink; *p* value is less than 0.05, if yellow; *p* value between 0.05 and 0.1 and the white means *p* value above 0.1

We also examined tumor intrinsic differences between immune subtypes, by investigating the association between overall survival and the expression of tumor intrinsic pathways in pan-cancer **(**Fig. 5A, Supplementary Table 6) and across the immune subtypes (Fig. 5C), and comparing the enrichment of tumor intrinsic pathways between the 6 immune subtypes (Fig. 5B). A wide variety of pathways were differentially enriched between immune subtypes. Myc targets, DNA repair and oxidative phosphorylation showed uniquely high enrichment in S4 compared to other groups. However, Wnt/beta-catenin and TGF-ß showed a similar enrichment pattern among immune subtypes with increased enrichment in S3 and S5 (Fig. 5B). Interestingly, most of the pathways show a mirrored expression level between S2 and S4; for example, S4 was significantly higher in the enrichment of TGF-ß and Barrier genes compared to S2, while p38 MAPK Signaling, ErbB2 ErbB3 Signaling, NOS1 Signature and SHC1/pSTAT3 Signatures were significantly highly enriched in S4 vs. S2 (*p* < 0.05). Within the immune subtypes, a significantly high association of some oncogenic pathways with worse prognosis is seen exclusively in S4 as mTORC1, Myc targets, NOS1, ERK5, PI3K AKT (Fig. 5C).

## Discussion

In this study we provide a comprehensive overview of the immunological landscape of pediatric tumors by dissenting immune-cancer interactions in relationship with clinical outcome. We previously showed that the Immunologic Constant of Rejection (ICR), a signature that captures the presence of active immune-mediated tumor rejection, has predictive and prognostic value in the context of adult cancer [28, 33]. We applied this signature in pediatric solid tumors to examine if it can also predict survival in pediatric tumors. We performed a per-cancer analysis of the 5 pediatric solid tumor types present in the TARGET cohort and found that the disposition of an immune active phenotype characterized by ICR high was associated with favorable prognosis in Osteosarcoma and high risk NBL without MYCN amplification. This association with survival is also observed when both adult and pediatric Osteosarcoma are evaluated together (C. Zhang et al. 2020).

When we attempted to disentangle the mechanism involved in tumor immune evasion, WNT/β-catenin was found to be associated with poor immune infiltration and cancer immunosurveillance in many adult tumors [59]. Here, we found an inverse correlation of WNT/β-catenin pathway enrichment with immune infiltration (ICR score) in OS with a prognostic value in the same cohort, in which the high enrichment of WNT/β-catenin pathway was associated with worse prognosis. In addition to the association with low immune infiltration and overall survival; a meta-analysis performed by Xie et al. suggested that overexpression of β-catenin is an indicator of metastasis for osteosarcoma patients [60]. Several clinical trials are ongoing applying the combination of immunotherapy with WNT/β-catenin signaling inhibitors for the treatment of various tumors (Y. Zhang and Wang 2020).

As seen in OS, we demonstrated that high risk NBL without MYCN amplification was associated with significantly higher immune infiltration and demonstrated the predictive value of the ICR signature in this sub-group of NBL. Recently, another signature focusing on effector genes (5 granzymes combined with perforin) that reflects the level of cytotoxic immune cell activity, showed correlation with survival in high risk NBL without MYCN amplification subgroup of NBL patients while not in the other groups [56].

In contrast, ICR gene expression showed an association with worse prognosis in Wilms tumor. This reverse association was also observed in adult kidney tumors like Kidney Renal Clear Cell Carcinoma (KIRC), Cervical Kidney renal papillary cell carcinoma (KIRP), in which a reverse association was observed between the ICR High classification and survival (Roelands et al. 2020). These combined observations convincingly show that the immune infiltrate in pediatric tumors does impact the pediatric cancer prognosis and resembles what was found in adult cancer,

We then deepened our analysis and demonstrated that solid pediatric cancer samples can be meaningfully immune subtyped, comparable to what was achieved in adult cancer (Thorsson et al. 2018). When applied in the pediatric setting, the same 5 representative signatures (INF-γ, TGF-β, Macrophages, Lymphocytes and Wound healing) identified in Thorsson et al. (Thorsson et al. 2018), were found to also be at the cores of the 5 main co-clustering modules of immune signatures in the combined TARGET cohorts. When we applied pan-cancer clustering on the TARGET samples based on the enrichment scores of these 5 representative signatures, despite the low number of tumor samples available, we obtained 6 robust immune subtypes. These displayed distinct immune characteristics and significant differences in overall survival rate even after adjusting for cancer type. Some of these immune subtypes share similar immune features with adult tumors, like the ‘Immunologically quiet’ subtype that is characterized by overall lower enrichment for T-cells and IFN-γ signature, or the ‘inflammatory subtype’, that is characterized by high Th1 enrichment, and which is associated with the best survival across all immune subtypes in pediatric and adult tumors.

High enrichment of the wound healing signature was associated with worse outcomes in adult tumors [41, 61], we observed the same in pediatric tumors in which the wound healing dominant subtype (S4) showed the worst survival across the pediatric immune subtypes. Wound healing enrichment is accompanied by elevated expression of angiogenic genes and high enrichment of proliferation signature. S4 also showed high proportions of Macrophages shown by CIBERSORTx (Supplementary Fig. 6), especially M2 macrophages (Supplementary Fig. 5).

In addition, high Th1 and low Th2 infiltration were observed in S2, while the reverse pattern was found in S4, which suggests the association of Th1 infiltration with favor prognosis in these pediatric tumors, the results that obtained in different adult cancer types [28, 33, 41, 62, 63]. Conversely, the Th2 was highly infiltrated in the wound healing subtype (S4) that is characterized by a worse prognosis and high proliferation rate, in agreement with the previous results in the adult cancer [41].

Furthermore, as we previously demonstrated, the Rhabdoid tumor is mainly dominated by S6, S5 and S4. These immune subtypes are suppressed by different mechanisms, either by: high Th2, Treg, immune checkpoints and exhaustion markers in S6, or high M2 macrophage presence in S5, or the increased expression of wound healing genes in S4. Looking at the ICR scores across the 6 immune subtypes (Fig. 2E), the highest ICR score was found in S6, S5 and S4. These observations highlight the importance of this more complex immune classification that captures the different characteristics of the immune microenvironment within the Rhabdoid tumor which the ICR score failed to capture.

When we look beyond the subtype at how underlying cancer intrinsic pathways interact with the immune system, we noted a significantly higher enrichment of DNA repair pathways in the S4 subtype compared to the other subtypes (Fig. 5B). DNA repair pathways defects in different cancers provide therapeutic opportunities to kill cancer cells without affecting normal cells taking advantage of the concept of synthetic lethality, which might be tested experimentally in these tumors [64–66].

Among the tumor intrinsic pathways that are highly enriched in S4 and associated with unfavorable prognosis is the mTORC1 signaling pathway. mTOR has critical roles in tumor progression, and mTOR complex 1 (mTORC1), is composed of mTOR bind to Raptor, GβL, and DEPTOR and could be inhibited by FDA approved drug Rapamycin [67]. We speculate that mTOR mechanisms, targeted by novel generation mTOR inhibitors [68], could be tested experimentally in patients with high mTROC1 signaling expression.

The most renowned mechanism of tumor immune evasion is of course the immunosuppressive checkpoint molecules that downregulate immune cell function in the aftermath of an infection. We show here that high expression of IDO1 and LAG3 could be observed in the wound healing immune subtype (S4) which may contribute to the poorer prognosis associated with this subtype. IDO1 is an immune regulatory gene that was shown to recruit and activate myeloid-derived suppressor cells through a Treg-dependent mechanism that contributed to aggressive tumor growth and poor response to T-cell targeted therapy [69]. Lymphocyte Activation Gene-3 (LAG3) on the other hand, has shown an immunomodulatory role through suppressing T-cell activation and cytokine secretion, and as a result, it maintains immune homeostasis. Both checkpoints could be considered as potential immunotherapeutic targets for patients classified as S4 and S1 in where LAG3 is highly expressed, in combination perhaps with other immune checkpoint inhibitors [70].

In addition, we found that other immune checkpoints like Cluster of Differentiation 70 (CD70) and TNF Superfamily Member 9 (TNFSF9, aka 4-1BB-L), are highly expressed in S4 compared to other immune subtypes, although these checkpoints are identified as co-stimulatory T cell immune checkpoints and are members of the tumor necrosis factor receptor (TNFR) family. CD70 has been shown to correlate with worse lung metastasis-free survival in primary human breast cancer isolated CSCs [71], and found to enhance the invasiveness of diffuse malignant mesothelioma of the pleura cells through MET–ERK axis activation in in-vitro experiments and in an immunodeficient mouse model [72]. It was shown that there is an unfavorable negative feedback function to downregulate inflammatory T cell responses obtained by T cell-derived CD70 through the upregulation of inhibitory immune checkpoints and caspase-dependent T cell apoptosis [73].

We also demonstrated that most of the immune checkpoints are highly expressed in S6 compared to the other subtypes, which could be further exploited as a therapeutic target in this subset.

One of the major limitations of this study is the relatively small sample size, which is however the reflection of the rarity of these tumors. Nevertheless, in spite of the small cohort, we observed significant differences across the subtypes suggesting a strong effect size for this immune-based classification, which could be clinically relevant.

## Conclusions

In conclusion, we demonstrated that extracranial solid pediatric tumors can be classified according to their immune disposition, unveiling unexpected similarities with adults’ tumors. Immunological parameters can be further explored to refine diagnostic and prognostic biomarkers and to identify potential immune-responsive tumors. Significant differences in the expression of immune checkpoints across the immune subtypes, and the different association of immune checkpoints with survival highlight the value of stratifying pediatric solid tumors into different immune phenotypes. This is the first pan-cancer immunogenomic analysis in children.

## Supplementary Information

**Additional file 1: Supplementary Fig. 1.** Correlation of ICR genes in pediatric tumors. (A) Pearson correlation heatmap of ICR genes in different pediatric tumors, immune regulator genes were colored in blue and immune active genes in red, positive correlation between genes represented by blue and negative correlation represented by red.

**Additional file 2: Supplementary Fig. 2.** The Immunologic constant of rejection. (A) Pan-cancer heatmap of ICR gene expression annotated by cancer types, and per-cancer clustering ICR High, medium and low. (B) Boxplot showing the distribution of ICR scores in high risk NBL with MYCN amplification, high risk NBL without MYCN amplification, and Intermediate and low risk NBL (the *p* value was calculated by two-tailed t-test). (C) Kaplan-Meier of overall survival for ICR High versus ICR low in Wilms tumor. (D) Kaplan-Meier event free survival curve for ICR High + medium (orange) versus ICR low (blue) in Osteosarcoma. (E) Kaplan-Meier of overall survival for ICR High versus ICR low in Rhabdoid tumor.

**Additional file 3: Supplementary Fig. 3.** Intrinsic oncogenic pathways and immune infiltration. (A) Heatmap of Pearson correlation between enrichment score (ES) of oncogenic pathways and ICR in pan-cancer, Osteosarcoma and high risk NBL without MYCN amplification. (B) Forest plot of HR of oncogenic pathways enrichment in Osteosarcoma (C) Forest plot of HR of oncogenic pathways enrichment in high risk NBL without MYCN amplification.

**Additional file 4: Supplementary Fig. 4.** Overall survival across Immune subtypes. (A) Kaplan-Meier overall survival curve for immune subtypes within Wilms tumor. (B) Kaplan-Meier overall survival curve for immune subtypes within Rhabdoid tumor. (C) Kaplan-Meier overall survival curve for immune subtypes within high risk NBL with MYCN amplification tumors. (D) Forest plot showing HRs (overall survival) of immune subtypes within high risk NBL with MYCN amplification tumors; S1, S2, S3, S5 versus S6 (E) Kaplan-Meier overall survival curve for immune subtypes within Intermediate and low risk NBL tumors.

**Additional file 5: Supplementary Fig. 5.** CIBERSORTx immune cells proportions across Immune subtypes. (A) Barplot of the median of proportions of CIBERSORTx immune cells in the 6 immune subtypes. (B) Boxplots of means of CIBERSORTx immune cells across the immune subtypes.

**Additional file 6: Supplementary Fig. 6.** CIBERSORTx immune cells proportions (Aggregate) across Immune subtypes. (A) Barplot of the median of proportions of aggregate CIBERSORTx immune cells in the 6 immune subtypes.

**Additional file 7: Supplementary Fig. 7.** Heatmap of enrichment scores of immune cells signatures. Left: Across the tumor types. Right: Across the immune subtypes.

**Additional file 8: Supplementary Table 1.** Immune subpopulation genesets.

**Additional file 9: Supplementary Table 2.** HR table for Immune sub-populations in pan-cancer.

**Additional file 10: Supplementary Table 3.** Correlation of 105 signatures in TARGET dataset.

**Additional file 11: Supplementary Table 4.** Multivariate overall survival Cox proportional hazards regression including Immune subtypes and clinical parameters in high risk NBL without mycn amplification.

**Additional file 12: Supplementary Table 5.** HR table for Immune checkpoints in pan-cancer.

**Additional file 13: Supplementary Table 6.** HR table for enrichment scores of oncogenic pathways Pan-cancer.

## Data Availability

Data are available in a public, open access repository. All data relevant to the study are included in the article or uploaded as online supplementary information using a Figshare repository. Scripts for analysis are available at Github repository https://github.com/Sidra-TBI-FCO/ILSPT. [REF:10.5281/zenodo.6605359]

## Abbreviations

CCSK: Clear Cell Sarcoma of Kidney
COG: Children’s Oncology Group
ES: Enrichment score
ICR: Immunologic constant of rejection
KIRP: Cervical Kidney renal papillary cell carcinoma
KIRC: Kidney Renal Clear Cell Carcinoma
K-M: Kaplan Meier
mDC: Macrophage-derived chemokine
MKI: Mitosis-Karyorrhexis Index
NBL: Neuroblastoma
OS: Osteosarcoma
RT: Rhabdoid tumor
TMB: Tumor Mutational Burden
WLM: Wilms tumor
