# Supplementary material for: The immune landscape of solid pediatric tumors
Source: J Exp Clin Cancer Res. 2022 Jun 11;41:199. doi: 10.1186/s13046-022-02397-z (PMC9188257; doi:10.1186/s13046-022-02397-z)

Supplementary figure1 . Pearson correlation of ICR genes in different pediatric tumors

A.

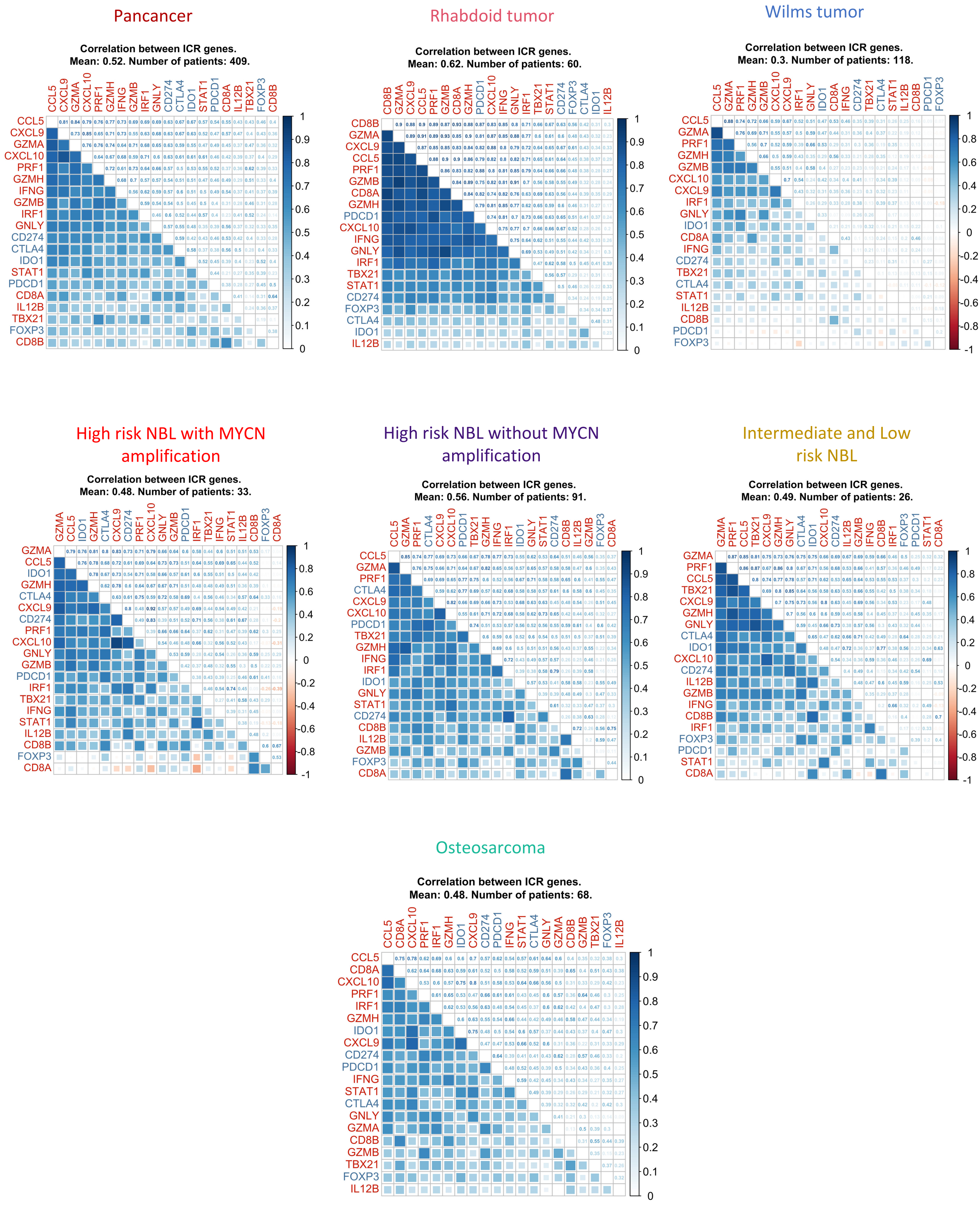

Supplement: Supplementary file 1 — Additional file 1: Supplementary Fig. 1. Correlation of ICR genes in pediatric tumors. (A) Pearson correlation heatmap of ICR genes in different pediatric tumors, immune regulator genes were colored in blue and immune active genes in red, positive correlation between genes represented by blue and negative correlation represented by red. [file 13046_2022_2397_MOESM1_ESM.pdf]
