# Supplementary figures and images for: The immune landscape of solid pediatric tumors
Source: J Exp Clin Cancer Res. 2022 Jun 11;41:199. doi: 10.1186/s13046-022-02397-z (PMC9188257; doi:10.1186/s13046-022-02397-z)

Supplementary figure 2. The Immunologic constant of rejection

A.

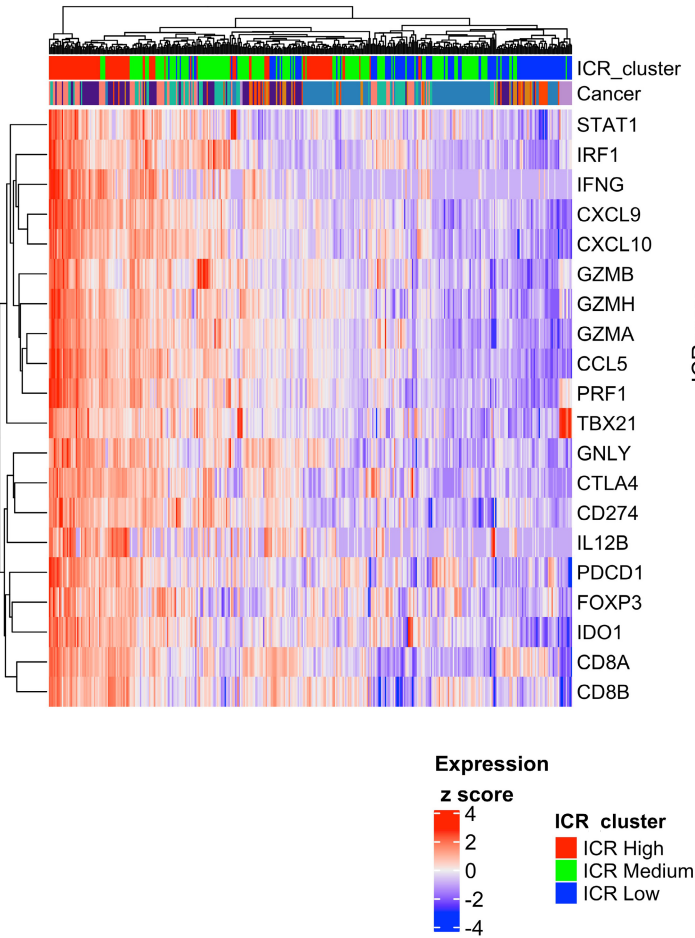

B.

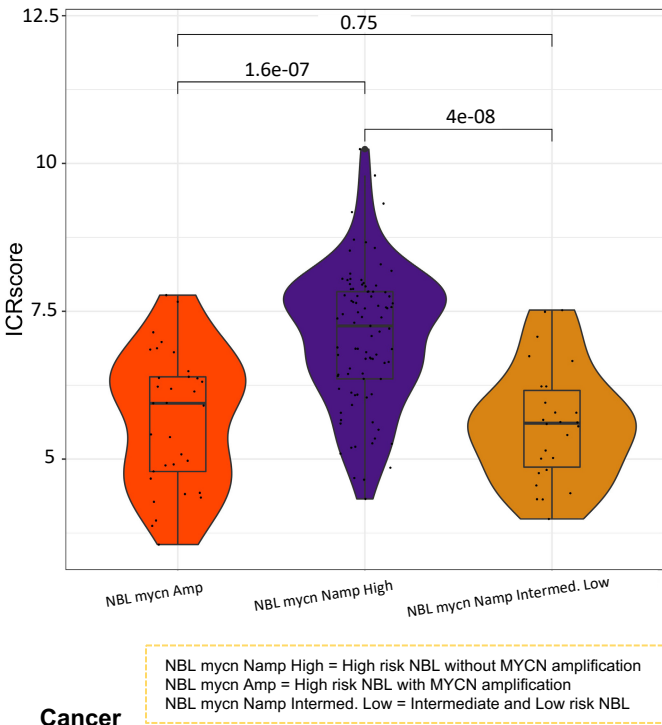

C.

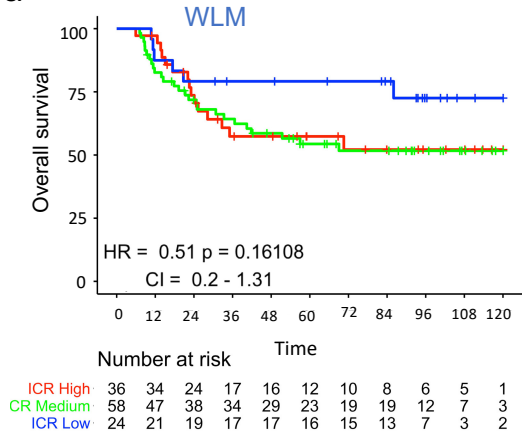

D.

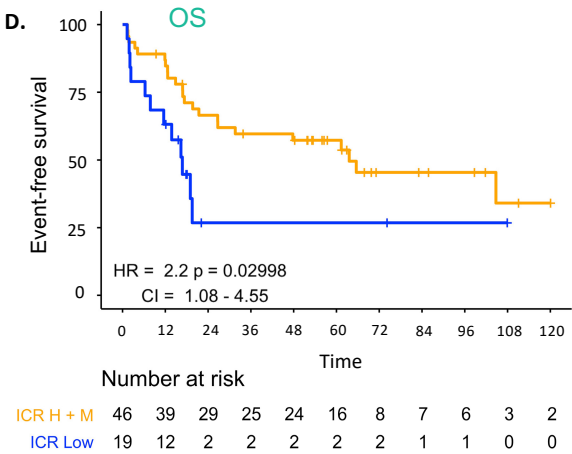

E.

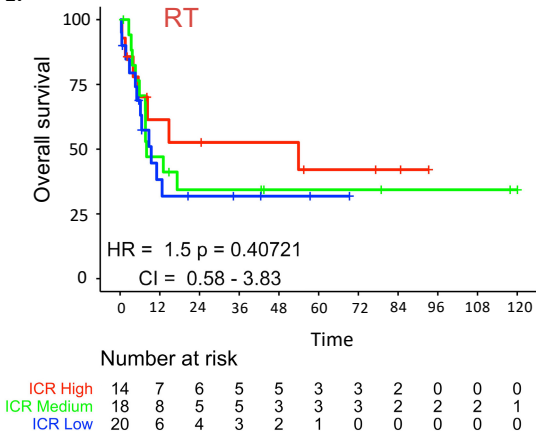

Supplement: Supplementary file 2 — Additional file 2: Supplementary Fig. 2. The Immunologic constant of rejection. (A) Pan-cancer heatmap of ICR gene expression annotated by cancer types, and per-cancer clustering ICR High, medium and low. (B) Boxplot showing the distribution of ICR scores in high risk NBL with MYCN amplification, high risk NBL without MYCN amplification, and Intermediate and low risk NBL (the p value was calculated by two-tailed t-test). (C) Kaplan-Meier of overall survival for ICR High versus ICR low in Wilms tumor. (D) Kaplan-Meier event free survival curve for ICR High + medium (orange) versus ICR low (blue) in Osteosarcoma. (E) Kaplan-Meier of overall survival for ICR High versus ICR low in Rhabdoid tumor. [file 13046_2022_2397_MOESM2_ESM.pdf]

Supplementary figure 3. Intrinsic oncogenic pathways across pediatric tumors

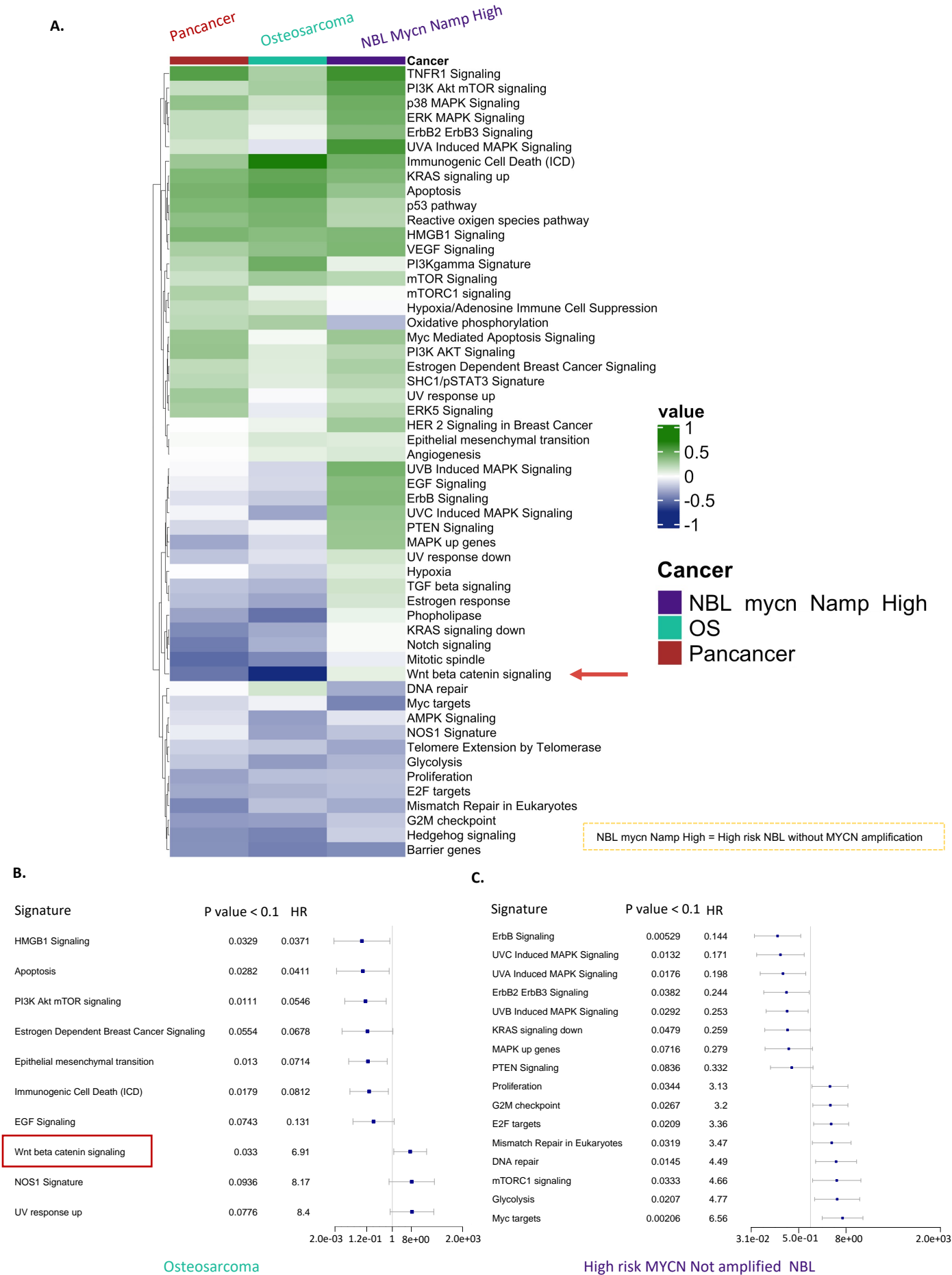

Supplement: Supplementary file 3 — Additional file 3: Supplementary Fig. 3. Intrinsic oncogenic pathways and immune infiltration. (A) Heatmap of Pearson correlation between enrichment score (ES) of oncogenic pathways and ICR in pan-cancer, Osteosarcoma and high risk NBL without MYCN amplification. (B) Forest plot of HR of oncogenic pathways enrichment in Osteosarcoma (C) Forest plot of HR of oncogenic pathways enrichment in high risk NBL without MYCN amplification. [file 13046_2022_2397_MOESM3_ESM.pdf]

Supplementary figure 4. Overall survival across Immune subtypes within cancer types

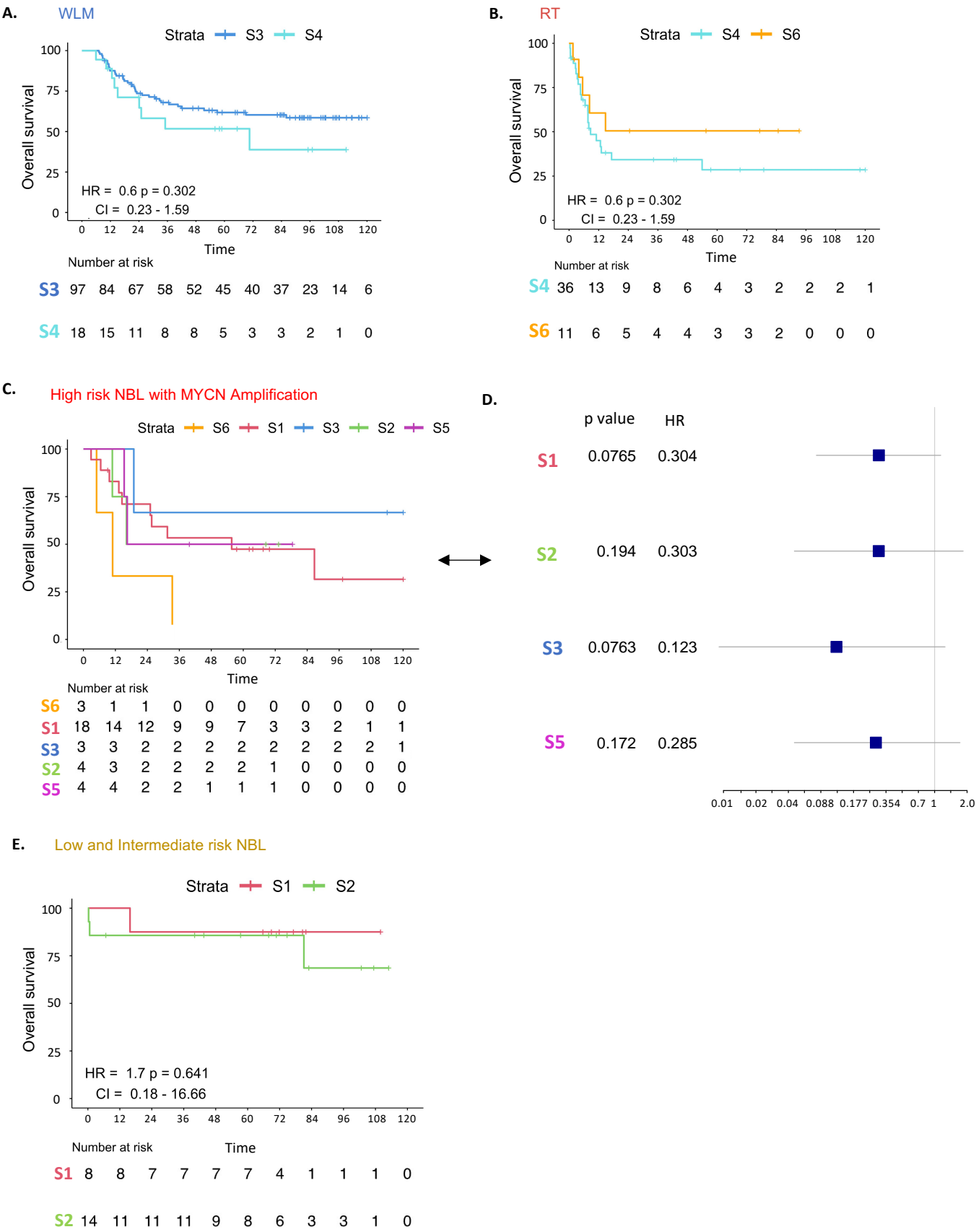

Supplement: Supplementary file 4 — Additional file 4: Supplementary Fig. 4. Overall survival across Immune subtypes. (A) Kaplan-Meier overall survival curve for immune subtypes within Wilms tumor. (B) Kaplan-Meier overall survival curve for immune subtypes within Rhabdoid tumor. (C) Kaplan-Meier overall survival curve for immune subtypes within high risk NBL with MYCN amplification tumors. (D) Forest plot showing HRs (overall survival) of immune subtypes within high risk NBL with MYCN amplification tumors; S1, S2, S3, S5 versus S6 (E) Kaplan-Meier overall survival curve for immune subtypes within Intermediate and low risk NBL tumors. [file 13046_2022_2397_MOESM4_ESM.pdf]

Supplementary figure 5. CIBERSORTx immune cells proportions across Immune subtypes

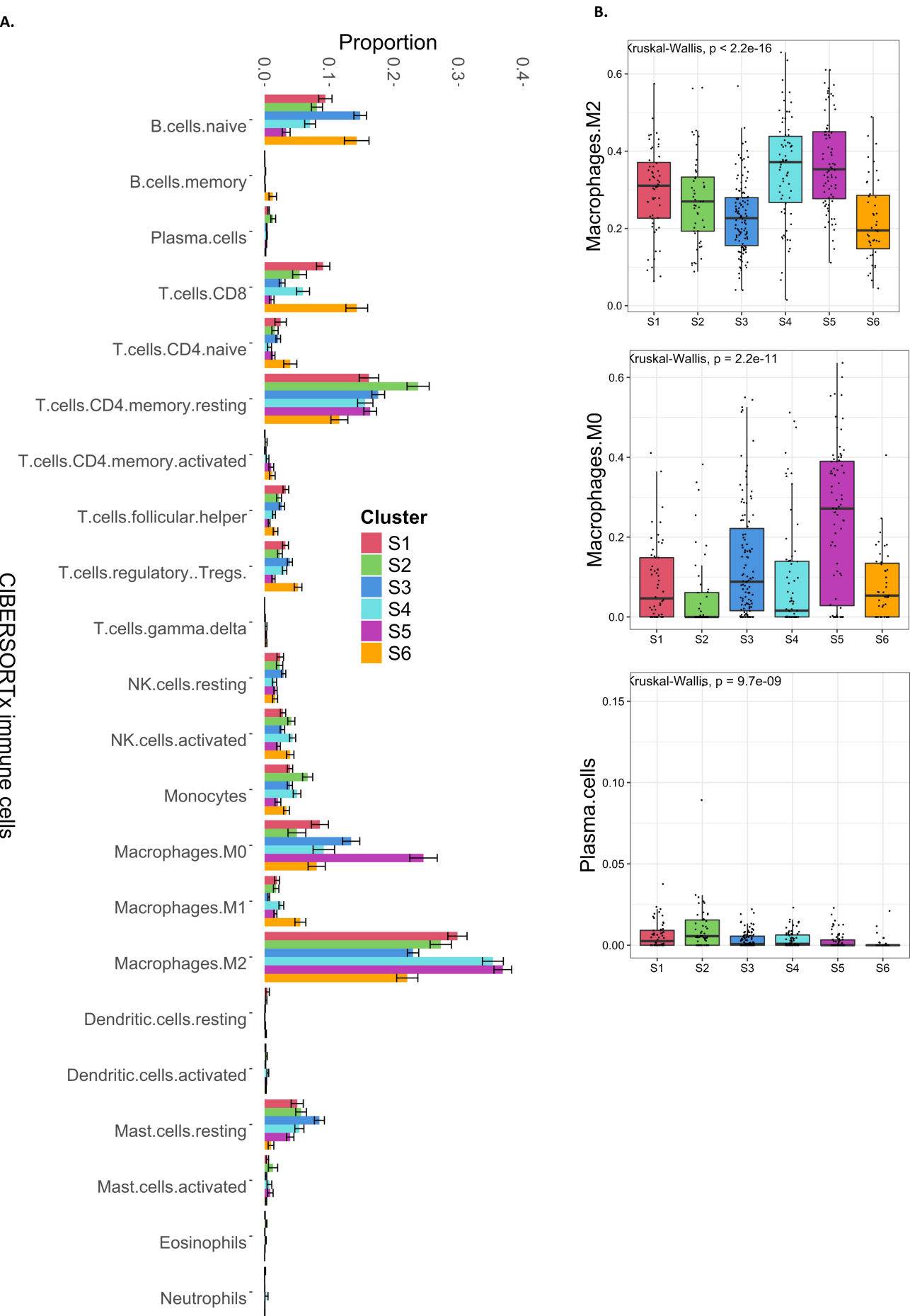

Supplement: Supplementary file 5 — Additional file 5: Supplementary Fig. 5. CIBERSORTx immune cells proportions across Immune subtypes. (A) Barplot of the median of proportions of CIBERSORTx immune cells in the 6 immune subtypes. (B) Boxplots of means of CIBERSORTx immune cells across the immune subtypes. [file 13046_2022_2397_MOESM5_ESM.pdf]

Supplementary figure 7. Heatmap of the enrichment scores of immune cells signatures.

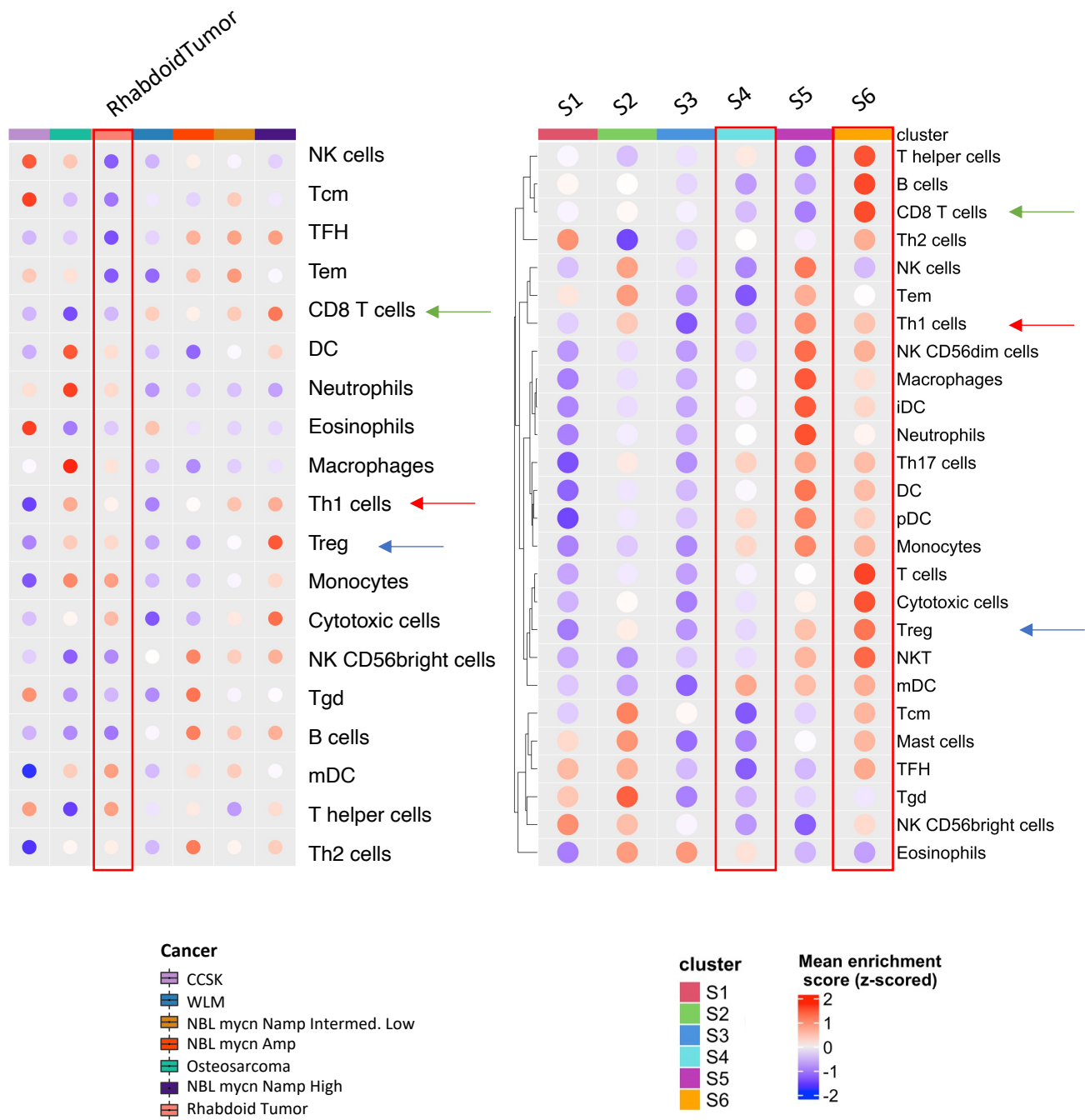

Supplement: Supplementary file 7 — Additional file 7: Supplementary Fig. 7. Heatmap of enrichment scores of immune cells signatures. Left: Across the tumor types. Right: Across the immune subtypes. [file 13046_2022_2397_MOESM7_ESM.pdf]
