# Supplementary material for: The immune landscape of solid pediatric tumors
Source: J Exp Clin Cancer Res. 2022 Jun 11;41:199. doi: 10.1186/s13046-022-02397-z (PMC9188257; doi:10.1186/s13046-022-02397-z)

Supplementary figure 6. CIBERSORTx immune cells proportions across Immune subtypes (Aggregate)

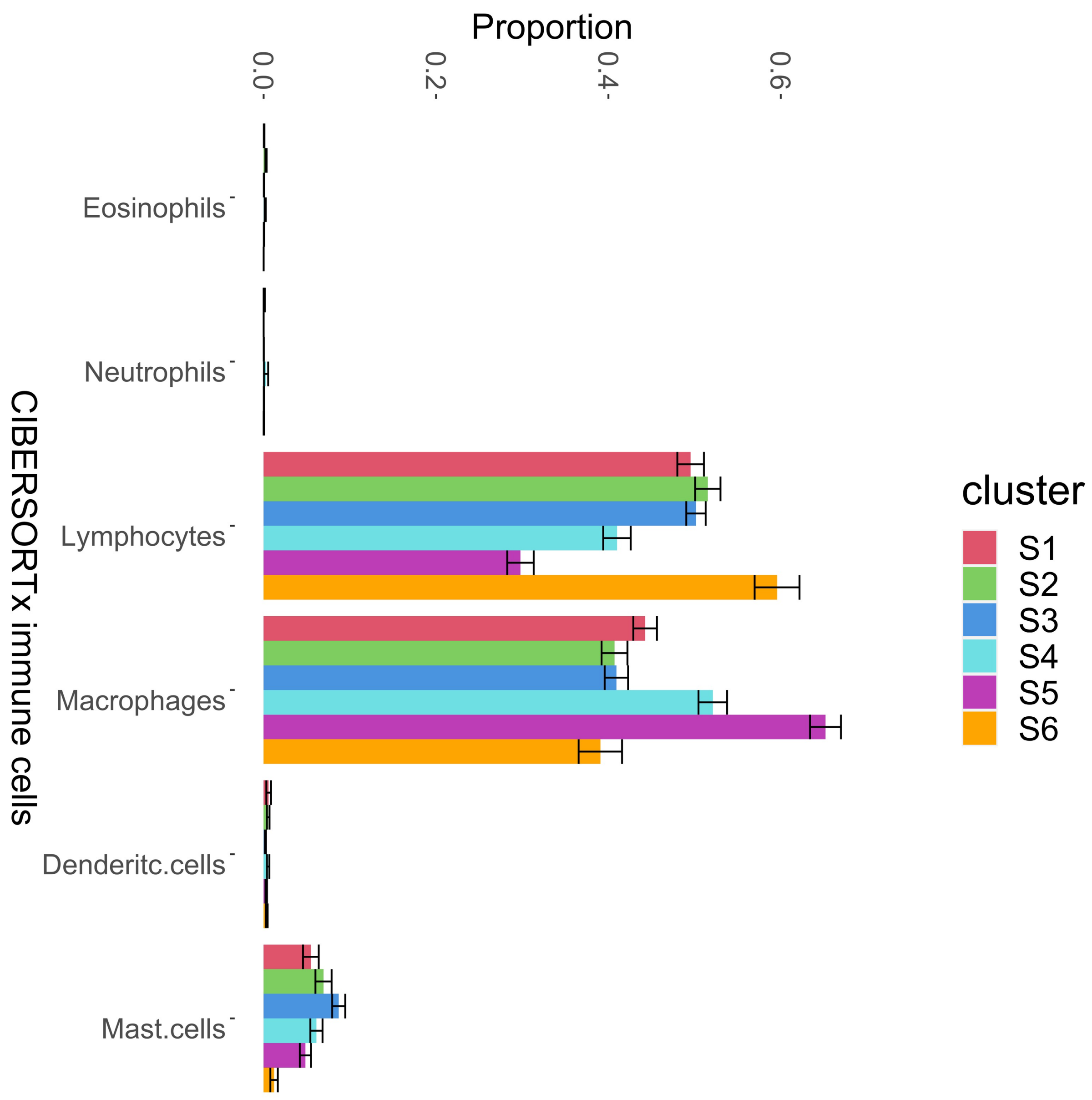

Supplement: Supplementary file 6 — Additional file 6: Supplementary Fig. 6. CIBERSORTx immune cells proportions (Aggregate) across Immune subtypes. (A) Barplot of the median of proportions of aggregate CIBERSORTx immune cells in the 6 immune subtypes. [file 13046_2022_2397_MOESM6_ESM.pdf]
